# Operationalizing Anti-Racism Accountability with Equitable Admissions in Nursing Education Accreditation

**DOI:** 10.1089/heq.2023.0099

**Published:** 2024-02-28

**Authors:** Alicia Swartz, Denise Dawkins, Claire Valderama-Wallace, Michelle DeCoux Hampton

**Affiliations:** ^1^ Department of Nursing, California State University, East Bay, Hayward, California, USA.; ^2^ The Valley Foundation School of Nursing, San Jose State University, San Jose, California, USA.; ^3^ Office of Research, Stanford Health Care, Palo Alto, California, USA.

**Keywords:** holistic admissions, health equity, anti-racism, nursing education, nursing, diversity

## Abstract

For decades, health professional organizations have recommended increased diversity in the workforce and education. To address persistent inequities in health care, the racial composition of the nursing workforce needs be congruent with the U.S. population. Without first addressing structural inequity in nursing education programs, the nursing profession cannot begin to address structural racism in health care. The lack of nursing student diversity is reflective of barriers in program admissions. This article is a call to nursing accreditation bodies to operationalize anti-racism to improve U.S. nursing workforce diversity by introducing accountability structures that require evidence-based holistic admission review and analysis of admission data to ensure that student cohorts are diverse across nursing programs, thereby ensuring a future workforce that reflects the diversity of the U.S. population.

The COVID pandemic resulted in a 34% nursing shortage in January 2022.^1^ The U.S. Bureau of Labor Statistics estimates that more than 275,000 additional nurses will be needed nationwide between 2020 and 2030.^2^ Despite the shortage, nursing schools rejected 80,521 qualified applicants in 2020.^3^ With the rising demand for nurses, selecting the most suitable candidates to deal with the challenging health care landscape is imperative.

It is well understood that increasing underrepresented racial and ethnic groups in health care training programs improves health care workforce diversity, thus improving health outcomes among underserved populations.^4,5^ Yet, a continued lack of diversity in nursing education programs perpetuates the lack of diversity in the U.S. nursing workforce. In 2017, 80.8% of Registered Nurses (RN) in the United States identified as white, with a modest increase to 80.6% in 2020.^6^ This disparity is a result of the historical and ongoing systematic exclusion of underrepresented racial and ethnic groups in nursing,^7,8^ and reflects a structural problem that contributes to the production and perpetuation of inequities and injustices seen in nursing, particularly the enduring systemic impact of whiteness and white supremacy.

As a strategy to improve health equity, several national organizations called for increased diversity in the nursing workforce. The American Association of Colleges of Nursing (AACN) launched the Building a Culture of Belonging in Academic Nursing program in January 2022 with Johnson & Johnson funding.^9^ The American Nurses Association similarly endorsed the AACN's position^10^ and the National Academy of Medicine published the “Future of Nursing 2020–2030: Charting a Path to Achieve Health Equity”^11^ that emphasized the importance of creating a diverse, equitable, and antiracist nursing workforce. Finally, the Institute of Medicine released the “The Future of Nursing” report in 2010^12^ with recommendations for an action-oriented blueprint to address diversity and structural inequities in nursing.

Despite multiple calls to advance health equity by improving racial diversity within the nursing workforce,^9–14^ little progress has been made.^15^ It is increasingly clear that, although advancing social justice, health equity, and addressing structural racism are described as expectations for nursing practice,^16^ the structural changes needed to operationalize anti-racism to bring about equitable change are not clearly defined. As a result, nurses lack the clear directive and accountability structures needed to advance health equity in patient care settings. The first critical step to reduce health inequities is addressing the structural impact of inequity and the lack of racial diversity among nursing faculty, staff, and students.

One of the key ways that nursing professional organizations standardize education across nursing programs is through accreditation. In general, accreditation systems provide regulatory oversight and guidance to standardize the quality of training needed to produce competent providers who are prepared to meet the current health care needs of the public.^17^ Nursing programs are subject to strict review by both university-wide and nursing-specific accrediting bodies. A number of organizations include diversity and equity education requirements in their accreditation standards, including the Accreditation Commission for Education in Nursing^18^ (an accrediting body for undergraduate and graduate nursing education) and the Commission on Collegiate Nursing Education (CCNE;^19^ an accrediting body for baccalaureate and graduate nursing education).

The CCNE Standards for Accreditation of Baccalaureate and Graduate Nursing Programs (2018)^20^ includes demonstration of an “institutional commitment to programming infrastructure, faculty qualifications, and workforce capacity” as a requirement for accreditation. While nursing programs provide admission policies as supporting documentation for their accreditation, we argue that accrediting organizations can take a more active role in holding institutions accountable for implementation and evaluation of evidence-based holistic admission practices and policies that are aligned with increasing student and workforce diversity.

In 2020, the AACN published recommendations for Holistic Admissions Review (HAR)^21^ that detailed inclusive recruitment and HAR strategies designed to broaden the criteria currently used to select applicants for nursing schools beyond traditional academic metrics (predominantly grade point average and standardized test scores) to include skills and competencies such as health-related work experience, language proficiency, and communication effectiveness. These skills are highly valuable in professional nurses; however, despite evidence that underrepresented racial and ethnic groups are disadvantaged by this approach to admissions, nursing schools continue to prioritize grade point average and standardized test scores as the determinant metrics for admission.^22^ Despite the Supreme Court's ruling that ended affirmative action in U.S. higher education,^23^ HAR processes can still be effective for increasing student diversity in colleges and universities without the use of racial quotas.^24^

In 2021, the AACN published The Essentials: Core Competencies for Professional Nursing Education,^16^ which defined HAR and cited their previous article recommending HAR, but did not suggest any specific guidance or expectation for nursing programs to adopt an evidence-based holistic admission process. Given that the AACN's recommendations for HAR are presented as optional, a standardized approach to implementing evidence-based holistic admissions practices nationally has not been established.^22,25^

The Essentials include subcompetencies related to inequities, systems-level thinking, diverse sources of evidence, inclusive team dynamics, and leadership to advance social justice and diversity. These, however, are intended to guide curricular development alone. The Essentials fall short of recommending structural change at the program level, which could influence the creation of diverse and inclusive student cohorts that not only reflect the composition of the local and national population but also have the potential to address health inequities.

Nursing has an imperative to address the lack of diversity in its education programs. However, the most powerful governing organizations have failed to implement accountability structures that require the use of evidence-based holistic admission practices and demonstration of success or failure in admitting diverse student cohorts. As a result, individual nursing educators are relied upon to resolve this systemic issue. Nursing program faculty and administration continue to use processes that are known to simply perpetuate the status quo and rely on the motivation and goodwill of predominantly white nursing educators with varying levels of understanding, expertise, or buy-in, to independently deconstruct systems of inequity.

Short-term initiatives designed to train current nurse educators to engage nursing students to reflect and recognize structural racism are insufficient. As a profession, nursing needs to emerge from under the veil of assumptions that those in positions of leadership and the prevailing structures they influence have the capacity to usher meaningful change without accountability. Instead, leaders must reimagine nursing as innovators and drivers of health equity across all health care systems.^26^ This must start with addressing the structures within admission policy and practices that lead to the lack of opportunities for underrepresented racial and ethnic groups to enter the nursing workforce.

Furthermore, these efforts must be paired with accountability systems that require measurement and reporting of structural inequities, such as the racial and ethnic composition of admitted cohorts. Program administrators must be transparent about the achievement of inclusion goals and explain efforts to adapt admission policies when they fail. Quantifying structural racism is one powerful way to operationalize anti-racism and address inequities by documenting the problem, increasing awareness, and using the data obtained to design interventions to address them.^26,27^

All governing nursing organizations, including State Boards of Nursing and accreditation organizations, must take an active role in restructuring recommendations for evidence-based holistic admission practices, equity outcome measurement, and reporting as requirements for accreditation, as a necessary and effective approach to improve racial and ethnic diversity in nursing education. These proposed accountability structures for admissions policies and practices will in turn strengthen efforts toward health equity and improve diversity in nursing to meet the current workforce needs of the U.S. health care system.

## Abbreviations Used

AACN: American Association of Colleges of Nursing
CCNE: Commission on Collegiate Nursing Education
HAR: Holistic Admissions Review

## References

[1] Ochieng N, Chidambaram P, Musumeci M. Nursing Facility Staffing Shortages During the COVID-19. Health Policy Research, Polling, and News; 2022. Available from: https://www.kff.org/c588989 [Last accessed: September 27, 2023].

[2] U.S. Bureau of Labor Statistics. Us Department of Labor Announces $80M Funding Opportunity to Help Train, Expand, Diversify Nursing Workforce; Address Shortage of Nurses. Employment and Training Administration; 2022. Available from: https://www.dol.gov/newsroom/releases/eta/eta20221003 [Last accessed: September 27, 2023].

[3] American Association of Colleges of Nursing (AACN). Student Enrollment Surged in U.S. Schools of Nursing in 2020 Despite Challenges Presented by the Pandemic. 2021. Available from: https://www.aacnnursing.org/News-Information/Press-Releases/View/ArticleId/24802/2020-survey-data-student-enrollment [Last accessed: September 27, 2023].

[4] Crear-Perry J, Correa-de-Araujo R, Lewis Johnson T, et al. Social and structural determinants of health inequities in maternal health. J Womens Health 2021;30(2):230–235; doi: 10.1089/jwh.2020.8882 PMC8020519 33181043

[5] Williams D, Cooper L. Reducing racial inequities in health: Using what we already know to take action. IJERPH 2019;16(4):606; doi: 10.3390/ijerph16040606 30791452 PMC6406315

[6] Smiley RA, Ruttinger C, Oliveira CM, et al. The 2020 National Nursing Workforce survey. J Nurs Regul 2021;12(1):S1–S96; doi: 10.1016/S2155-8256(21)00027-2

[7] Carnegie ME. The Path We Tread: Blacks in Nursing, 1854–1990. 2nd ed. National League for Nursing Press: New York, NY; 1991.

[8] American Nurses Association (ANA). Our Racial Reckoning Statement. 2022. Available from: https://www.nursingworld.org/~4a00a2/globalassets/practiceandpolicy/workforce/racial-reckoning-statement.pdf [Last accessed: September 27, 2023].

[9] American Association of Colleges of Nursing (AACN). Enhancing Diversity in the Nursing Workforce. Fact Sheet. 2023. Available from: https://www.aacnnursing.org/Portals/0/PDFs/Fact-Sheets/Enhancing-Diversity-Factsheet.pdf [Last accessed: September 27, 2023].

[10] American Nurses Association (ANA). Establishing a Culturally Competent Master's and Doctorally Prepared Nursing Workforce. 2009. Available from: https://www.nursingworld.org/practice-policy/nursing-excellence/official-position-statements/id/establishing-a-culturally-competent-masters-and-doctorally-prepared-nursing-workforce [Last accessed: September 27, 2023].

[11] Committee on the Future of Nursing 2020–2030; National Academy of Medicine; National Academies of Sciences, Engineering, and Medicine. The Future of Nursing 2020–2030: Charting a Path to Achieve Health Equity. (Wakefield MK, Williams DR, Menestrel SL, et al. eds). National Academies Press: Washington, DC; 2021; doi: 10.17226/25982 34524769

[12] Bleich MR. IOM report, the future of nursing: Leading change, advancing health: Milestones and challenges in expanding nursing science. Res Nurs Health 2011;34(3):169–170; doi: 10.1002/nur.20433 21520144

[13] Institute of Medicine (IOM); Committee on the Robert Wood Johnson Foundation Initiative on the Future of Nursing, at the Institute of Medicine. The Future of Nursing: Leading Change, Advancing Health. National Academies Press: Washington, DC; 2011; doi: 10.17226/12956

[14] Robert Wood Johnson Foundation. The Changing Face of Nursing: Creating a Workforce for an Increasingly Diverse Nation. Robert Wood Johnson Foundation; 2016. Available from: https://www.rwjf.org/en/insights/our-research/2016/01/the-changing-face-of-nursing.html [Last accessed: September 27, 2023].

[15] National League for Nursing. NLN Releases New Survey Results of Nursing Schools & Programs Showing Persistent Challenges to Addressing the Nursing Shortage: Little Progress Shown in Faculty & Student Diversity or Expansion of Capacity to Admit Qualified Applicants. 2023. Available from: https://www.nln.org/detail-pages/news/2023/09/25/nln-releases-new-survey-results-of-nursing-schools-programs-showing-persistent-challenges-to-addressing-the-nursing-shortage [Last accessed: September 27, 2023].

[16] American Association of Colleges of Nursing (AACN). The Essentials: Core Competencies for Professional Nursing Education. American Association of Colleges of Nursing (AACN); 2021. Available from: https://www.aacnnursing.org/Portals/42/AcademicNursing/pdf/Essentials-2021.pdf [Last accessed: September 27, 2023].

[17] Frank JR, Taber S, van Zanten M, et al. The role of accreditation in 21st century health professions education: Report of an International Consensus Group. BMC Med Educ 2020;20(S1):305; doi: 10.1186/s12909-020-02121-5 32981519 PMC7520947

[18] Accreditation Commission for Education in Nursing (ACEN). ACEN 2023 Program Guidelines. 2023. Available from: https://www.acenursing.org/2023-resources/ACEN-2023-Program-Guidelines.pdf [Last accessed: September 27, 2023].

[19] Commission on Collegiate Nursing Education (CCNE). Commission on Collegiate Nursing Education (CCNE). 2023. Available from: https://www.aacnnursing.org/ccne-accreditation [Last accessed: September 27, 2023].

[20] Commission on Collegiate Nursing Education (CCNE). Standards for Accreditation of Baccalaureate and Graduate Nursing Programs. 2018. Available from: https://www.aacnnursing.org/Portals/42/CCNE/PDF/Standards-Final-2018.pdf [Last accessed: September 27, 2023].

[21] American Association of Colleges of Nursing (AACN). Promising Practices in Holistic Admissions Review: Implementation in Academic Nursing. American Association of Colleges of Nursing (AACN); 2020. Available from: https://www.aacnnursing.org/Portals/42/News/White-Papers/AACN-White-Paper-Promising-Practices-in-Holistic-Admissions-Review-December-2020.pdf [Last accessed: September 27, 2023].

[22] Hampton MD, Dawkins D, Rickman Patrick S, et al. Holistic admissions review integration in nursing programs. J Nurs Educ 2022;61(7):361–366; doi: 10.3928/01484834-20220610-01 35858128

[23] Supreme Court of the United States. No. 20–1199. Students for Fair Admissions, Inc., v. President and Fellows of Harvard College. 2023. Available from: https://www.supremecourt.gov/opinions/22pdf/20-1199_hgdj.pdf [Last accessed: September 27, 2023].

[24] Hampton MD, Apen LV. Impact of rubric weight on holistic admissions for underrepresented students in nursing. Teach Learn Nurs 2022;17(4):344–349; doi: 10.1016/j.teln.2022.07.007

[25] Hampton MD, Dawkins D, Rickman Patrick S, et al. Nursing program admission barriers in the United States: Considerations for increasing Black student enrollment. Nurse Educ 2022;47(1):19–25; doi: 10.1097/NNE.0000000000001071 34310420

[26] McLemore MR. Using retrofit, reform, and reimagine to advance toward health equity. J Perinat Neonatal Nurs 2022;36(2):99–102; doi: 10.1097/JPN.0000000000000639 35476759

[27] Groos M, Wallace M, Hardeman R, et al. Measuring inequity: A systematic review of methods used to quantify structural racism. J Health Disparities Res Pract 2018;11(2, Article 13):190–206.

